# Online correction of intrafraction motion during volumetric modulated arc therapy for prostate radiotherapy using fiducial‐based kV imaging: A cohort study quantifying the frequency of shifts and analysis of men at highest risk

**DOI:** 10.1002/acm2.14603

**Published:** 2025-01-17

**Authors:** Lucas M. Serra, Tianming Wu, Mark C. Korpics, Kamil Yenice, Stanley L. Liauw

**Affiliations:** ^1^ Department of Radiation and Cellular Oncology University of Chicago Medical Center Chicago Illinois USA

**Keywords:** IFM, intrafraction motion, onboard imaging, prostate cancer, shift correction

## Abstract

**Background:**

Various methods exist to correct for intrafraction motion (IFM) of the prostate during radiotherapy. We sought to characterize setup corrections in our practice informed by the TrueBeam Advanced imaging package, and analyze factors associated with IFM.

**Methods:**

132 men received radiation therapy for prostate cancer with a volumetric modulated arc therapy technique. All patients underwent planning CT immediately following transrectal placement of 3 fiducial markers. The most common RT course was 20 fractions (range: 17–44). Triggered kV images were acquired every 15 seconds over 2–3 full arcs using an onboard imaging system. IFM correction was considered when if any two fiducial markers in a single kV image were observed to be outside beyond a 3 mm tolerance margin. A manual 2D/3D match was performed using the fiducial markers from the single triggered kV image to obtain a suggested couch shift. Shift data for three (x, y, z) planes were extracted from the record and verify system and expressed as a single 3‐dimensional translation. Shift percent (SP) was defined as the number of instances of an intrafraction correction divided by the total number of fractions for a given patient.

**Results:**

Over 2659 fractions of radiation, IFM was observed and corrected for 582 times across 463 (17%) fractions, and at least one shift was made over the course of treatment in 77% of men. Univariate analysis revealed that larger rectal volume or width, smaller prostate volume, and use of ADT were associated with SP > 20% (p < 0.05). Men with a rectal width >3.6 cm were more likely to have IFM corrected (SP > 20% 47% vs 18%, p = 0.0016). On multivariate analysis, only rectal volume and width were associated with IFM.

**Conclusions:**

In this cohort study, 17% of fractions were interrupted to apply at least one couch shift. Men treated with shorter courses of therapy, such as stereotactic body radiation therapy, or men at high risk for IFM (e.g. larger rectal size) may warrant more careful consideration regarding the implications of IFM.

## 1 INTRODUCTION

Although newer technology has facilitated an improvement in conformality and reduction in treatment time, setup error remains an important factor in the success of radiation therapy for prostate cancer. Intrafraction motion (IFM), defined as internal organ motion of bladder, rectum and prostate or patient movement during an individual fraction of external beam radiation therapy, has been characterized in several studies, and many approaches exist to account for IFM.^1^ As treatments become increasingly precise with tighter planning target volume (PTV) margins, along with moderate to highly hypofractionated dose regimens, the impact of IFM on delivered dose accuracy may increase.^2^ We recently published a feasibility report using a commercially available triggered imaging technique to monitor and correct for IFM (Varian Truebeam Advanced IGRT & Motion Package, Varian Medical Systems, Palo Alto, CA, USA).^3^ During treatment, this system acquires kV images at user‐specified intervals with the readily available onboard imager (OBI), and determines the location of implanted fiducial markers. It subsequently decides whether markers are within a pre‐specified tolerance and alerts the radiation therapist to consider a positional correction by shifting the treatment couch. In the current work, we sought to quantify the degree of IFM observed in a larger cohort, and analyze clinical factors associated with IFM.

## 2 MATERIALS AND METHODS

One hundred and thirty‐two men with prostate cancer were treated with radiation therapy (RT). All patients underwent a planning CT scan immediately following transrectal placement of three prostatic fiducial markers (Gold Anchor, Naslund Medical, Stockholm, Sweden). Patients were immobilized via an alpha‐cradle (Smithers Medical Products Inc, North Canton, OH) or Vac‐Lok vacuum bag (CQ Medical, Avondale, Pennsylvania, USA). A catheter was used to drain the bladder and 120 cc of contrast was instilled into the bladder in tandem with a urethrogram prior to the acquisition of images. Related to the placement of fiducial markers immediately prior to CT simulation, patients were instructed to use a fleet enema prior to simulation. Patients were treated with volumetric modulated arc therapy (VMAT), most commonly to 60 Gy in 20 fractions (range: 17–44) to the prostate, with elective inclusion of seminal vesicles and pelvic lymph nodes at clinician discretion. Androgen deprivation therapy (ADT) consisting of LHRH‐agonist and oral anti‐androgen was given in a neoadjuvant (2 month) and concurrent (1–2 month) setting, with adjuvant LHRH‐agonist continuing for a total of 6–28 months, depending on patient and clinical factors. An anisotropic PTV margin of 8 mm laterally, 6 mm superiorly/inferiorly, and 5 mm posteriorly was applied to the prostate clinical target volume for setup error. CT slice thickness was 2 mm and the lateral area of fiducials markers was 3.2 mm^2^. During treatment planning, fiducial markers were delineated and reference points were created at their centers of mass.

In each treatment fraction, an orthogonal kV image pair was used to setup patients for treatment by prioritizing the matching of implanted fiducial marker locations. If a large discrepancy between markers and bony anatomy was noticed, a Cone beam CT (CBCT) would be employed to assess soft tissue alignment. CBCTs were also obtained once or twice weekly prior to treatment to evaluate soft tissue alignment. During treatment, triggered kV images were acquired every 15 s (5%–10% through a fraction for a typical treatment consisting of 2–3 full arcs) using the onboard imaging system. On each image, the system detected the markers and compared their locations to the reference: a 3 mm radius circle around the fiducial center points. The color of the circles would change from green to red to alert radiation therapists that a detected marker was approximated to be beyond the 3 mm tolerance. If one single marker was outside tolerance on a single image, treatment would continue; if the same single marker was outside tolerance on two consecutive images, the therapist would apply a beam hold until the next triggered kV to reevaluate. If the position of the marker was still outside tolerance, or if at any point two markers were outside tolerance, the therapist would perform a 2D/3D match based on the single image and apply correction using translational couch shifts in vertical, longitudinal or lateral directions. If all three markers were beyond tolerance, the therapist would stop treatment, repeat the orthogonal pair kV‐kV matching and apply necessary shifts. Ultimately, the treating therapists had full discretion to hold treatment based on the position of markers with respect to the perceived location out of tolerance, and shifts smaller than 3 mm were allowed. For this study, three‐dimensional couch shifts were extracted from the record and verify system after any correctional shifts. Their magnitudes were calculated to assess the degree of IFM and expressed as a single translation (i.e., vector) using the square root of the sum of squares of the individual three‐dimensional shifts. Of note, in men who had pelvic lymph nodes treated with the prostate, we chose to prioritize setup to the prostate, despite the possibility for there to be a mismatch in setup with the bony anatomy due to prostate motion, based on the low chance for meaningful clinical impact related to this setup variance.^4^

Patient and clinical information was extracted from the patient's medical record including body mass index (BMI), use of ADT, and volumes of prostate, bladder, and rectum at simulation. Contouring of the prostate and organs‐at‐risk was performed in accordance with the RTOG normal male pelvis contouring atlas.^5^ Rectal width was measured as the transverse dimension of the rectum at the mid‐gland of the prostate on simulation CT.

All tests were performed in R statistical software (R version 4.3.1) and JMP® (Version 17, SAS Institute Inc., Cary, NC, 1989–2023) and were two‐tailed. Rectal volume and width, bladder volume, prostate volume and BMI were divided into quartiles for analysis. Shift percentage (SP) was defined as the number of intrafraction corrections divided by the total number of fractions for a given patient. The upper quartile (SP > 20%) was chosen as the endpoint. Our Institutional Review Board (IRB) approved this study (#14‐934A).

## 3 RESULTS

**FIGURE 1:**
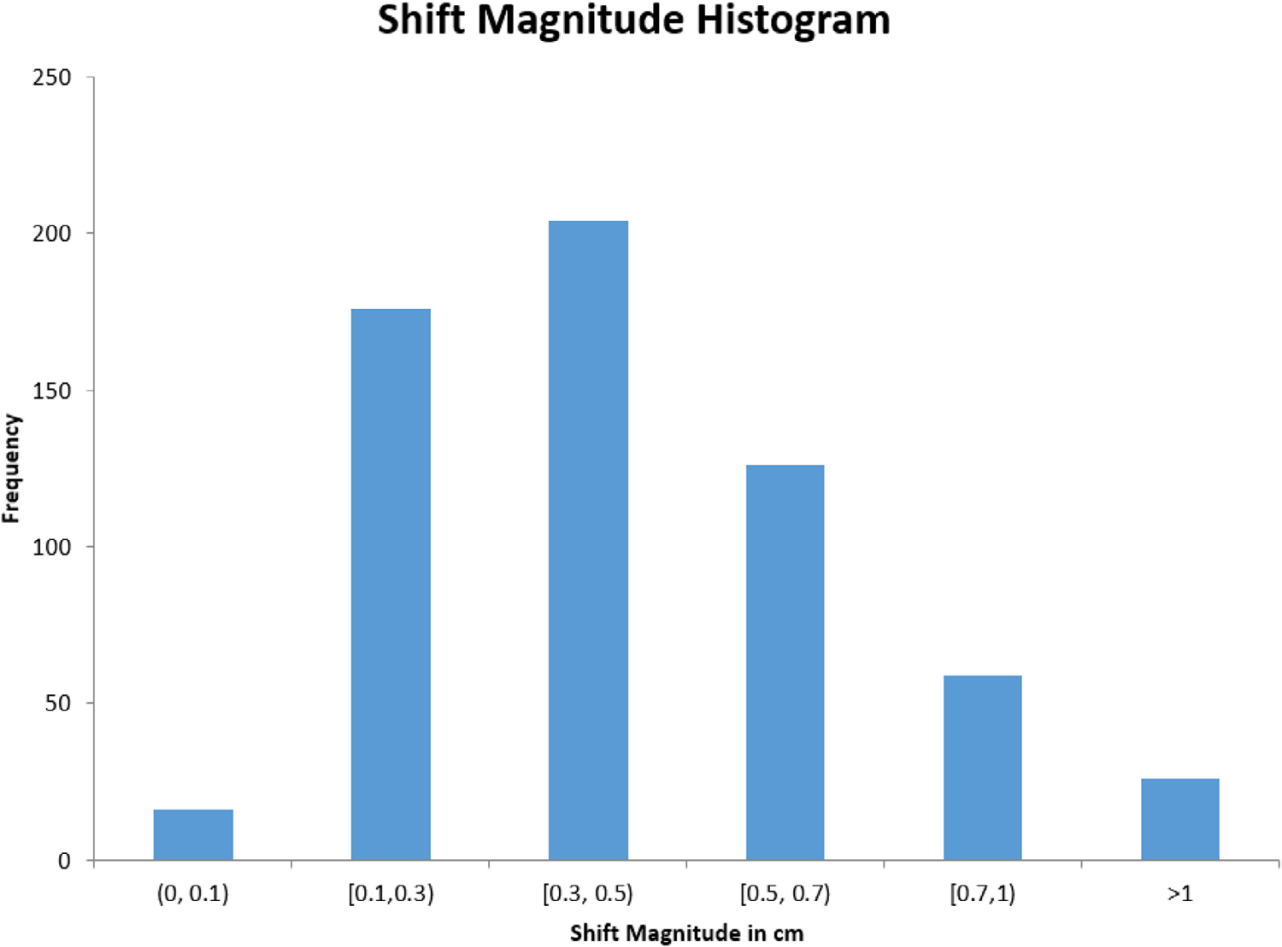
Histogram demonstrating the magnitude of intrafraction shifts across 2659 treatment fractions. Each bar shows a shift range above the lower boundary and up to the upper boundary (e.g. >0.3 cm and up to 0.5 cm).

Table 1 summarizes the clinical characteristics of the cohort. Across 2659 fractions of RT, IFM correction was performed 582 times in 463 (17%) fractions. 101/132 patients (77%) had at least one shift during their treatment course while 48/132 patients (36%) had shifts with an average magnitude of at least 5 mm. The median intrafraction shift was 3.6 mm (range, 0–2.4 cm; IQR, 1.5–5.4 mm). For patients who had a shift, Figure 1 depicts a histogram of the frequency of raw shift magnitudes on a per fraction basis. Figure S1 shows the frequency of shift magnitude in separate x, y, z planes.

**TABLE 1:** Patient characteristics (*n* = 132).

	Number (%) or Median (IQR)
Race	
Black	84 (64%)
White	36 (27%)
Asian	4 (3%)
Two or more races	2 (2%)
Unknown	6 (5%)
Hispanic or Latino	
No	120 (91%)
Yes	6 (5%)
Unknown	6 (5%)
T stage	
T1	74 (56%)
T2	31 (23%)
T3	18 (14%)
T4	2 (2%)
Unknown	7 (5%)
NCCN risk group	
Intermediate risk	51 (39%)
High risk	70 (53%)
Metastatic	11 (8%)
Number of radiation fractions	20 (20–20)
Number of radiation treatment arcs	
2	59 (45%)
3	62 (47%)
4	11 (8%)
Received hormonal therapy	94 (71%)
Prostate volume (cc)	41 (32–62)
Body mass index	29 (25–33)
Rectal volume (cc)	87 (68–111)
Rectal width (cm)	3.1 (2.7–3.6)
Bladder volume (cc)	181 (152–231)

Abbreviations: IQR, interquartile range; NCCN, National Comprehensive Cancer Network.

Univariate analysis revealed that larger rectal volume or width, smaller prostate volume, and use of ADT were associated with SP > 20% (*p* < 0.05). Prostate volume and ADT use were correlated; the median volume with and without ADT was 45 cc versus 70 cc, respectively (*p* = 0.0004). Men with rectal width in the top quartile (>3.6 cm) were more likely to have IFM corrected (SP > 20% was 47% vs. 18%, *p* = 0.0016), and similarly men with a rectal volume in the top quartile (> 112 cc) were more likely to have SP > 20% (44% vs. 19%, *p* = 0.0067). Bladder volume and BMI were not associated with SP > 20%. Multivariate analysis (MVA) including rectal width, ADT, and prostate size demonstrated a significant association with rectal width and SP > 20% (top quartile vs. otherwise, OR 4.14, *p* = 0.0015), but not for ADT use (*p* = 0.13) or prostate volume (*p* = 0.90, Table 2). A separate model including an interaction term for ADT*prostate size did not significantly change the model output (data not shown). Given the trend observed with ADT use and SP > 20%, the relationship between rectal width and SP > 20% was evaluated in subsets of men with and without ADT. In this exploratory subset analysis, the association of increasing rectal width and SP was stronger in men on ADT (Figure S2).

**TABLE 2:** Multivariate analysis for > 20% shift percent endpoint.

	Odds ratio (95% confidence interval)	*p* value
Rectal width > 3.6 cm (top quartile vs. other)	4.14 (1.72–9.96)	0.0015
Hormonal therapy (yes vs. no)	2.22 (0.76–6.46.)	0.13
Prostate volume, cc (continuous)	1.00 (0.99–1.02)	0.90

## 4 DISCUSSION

In this cohort study evaluating IFM of the prostate in men treated with VMAT for prostate cancer, we found that 17% of fractions were interrupted to apply at least one couch shift. The magnitude of IFM was 3.6 mm on average, and was ≥5 mm in 36% of patients thereby potentially resulting in underdosing of the intended clinical target volume that had been expanded by 5–8 mm for set up error. Clinicians should be aware of this potential for dosimetric uncertainty at the periphery of the treatment volume, and either correct for IFM in real time, adjust the PTV accordingly, or accept the risk of underdosing the target. Men treated with shorter courses of therapy, such as stereotactic body radiation therapy, or men at high risk for IFM (e.g. larger rectal size, or men receiving ADT) may warrant more careful consideration regarding the implications of IFM.

The degree of IFM observed in this cohort is larger than other studies that report an average shift magnitude of 1–2 mm and 28% of men having a >3 mm shift.^6^, ^7^, ^8^ This may in part be due to the fact that some prior studies only report the X, Y, Z components and not the vector of the shift as we have. IFM can result in underdosing of the clinical target volume, and could be particularly impactful with tight PTV margins^9^ or extreme hypofractionation. This degree of motion can be addressed with PTV margins of 5–8 mm; however, larger margins are not without consequence as a 5 mm reduction in separation between the prostate and rectum increases rectal V70 Gy by ∼50% when prescribing 78 Gy to the PTV.^10^ Several publications have examined the dosimetric consequences of IFM using motion monitoring systems in prostate radiotherapy. Li et al. analyzed prostate motion in 1267 tracking sessions using radiofrequency transponders (Calypso System by Varian Medical Systems, Palo Alto, CA) across 35 patients receiving prostate radiotherapy and found that a 2 mm PTV margin was not significantly different from a 5 mm PTV margin for conventionally fractionated radiotherapy plans when using the Calypso System.^11^ A similar study used radiofrequency transponders to continuously monitor prostate position with a 2 mm displacement threshold in 89 patients receiving SBRT. Their analysis found that without continuous monitoring, IFM would have resulted in roughly 10% of patients not meeting a PTV D95 of >90%.^12^ More recently, a study of 20 patients receiving prostate SBRT used pre‐and post‐ treatment CBCT alongside triggered KVs to fiducial markers every 3 s reported on volume coverage. The 95% isodose volumes covered the prostate volume in all but two fractions and the 98% isodose volumes in all but eight (4%) fractions. Notably, 90% of shifts were below the authors’ 3 mm deviation limit.^13^ As evidenced in Figure 1, a number of the shifts in our study had a magnitude below our threshold limit of 3 mm. This is likely a consequence of both the innate tolerance of the system as well as the operator‐dependent aspect of image review and shift implementation. These <3 mm shifts may be used to minimize misalignment of the other two fiducial markers or when shifts approach but do not quite surpass the 3 mm threshold.

This work is one of the largest studies of prostatic IFM treated with VMAT. (1) Our use of triggered kV imaging during treatment offers a periodically updated (every 15 s) and more accurate view of IFM compared to some prior studies exploring clinical factors that used pre and post‐treatment CBCT.^14^, ^15^ We analyzed a diverse range of clinical parameters as done by others^15^, ^16^ following a standardized procedure for planning including rectal enema for fiducial marker placement and simulation.^17^ However, there was no daily rectal prep during treatment itself and patients were not given specific instructions in this regard. If the rectum was significantly different from their simulation, patients were not instructed to evacuate. In our study, only rectal width and size were strongly correlated with SP. Adamson et al. examined pre‐ and post‐treatment CBCTs in 20 prostate cancer patients and also found that rectal filling was associated with prostate IFM as measured by intrafraction kV fluoroscopy. Moreover, gas volume was correlated with the maximum displacement during radiotherapy delivery.^14^ Another small study of 15 prostate cancer patients compared pre‐ and post‐treatment CBCTs to the initial planning CT to calculate IFM and found that motion increased with hormone therapy, larger bladder filling, and larger changes in bladder filling compared to the initial scan.^15^ The authors also found that rectal filling was predictive of the direction of prostate motion but not the magnitude of displacement, and they attributed this finding to the adequacy of their rectal preparation protocol. Shelton et al. analyzed 37 patients and found that longer session times were the strongest predictor of IFM.^18^ Meanwhile, a more recent study of 331 men examined patient factors including rectal, bladder, and prostate volumes and found no relation with IFM during CyberKnife treatment.^19^ Finally, IFM has been examined using MRI‐based linear accelerators with two studies demonstrating the feasibility of automatic prostate tracking based on high‐ quality 3D cine‐MR imaging.^20^, ^21^

While we did not find ADT use to be associated with SP on MVA, it did appear to influence the correlation between rectal width with IFM in a linear fit model. It is possible that a reduction in prostate volume, a well described phenomenon during ADT,^22^, ^23^, ^24^, ^25^ resulted in a larger relative impact of rectal filling on IFM. However, prostate size was not found to be associated with IFM, so another possible explanation is that our exploratory findings are influenced by the smaller number of patients and events of subset analysis. We cannot exclude the potential for an alternative explanation, such as ADT causing IFM related to differential rectal filling through altered peristalsis.

We acknowledge a few limitations in our study. The findings of this single institution study may not be generalizable to other patient cohorts, including men who do not undergo enema prior to fiducial placement on the same day of simulation. Our prior work has shown that there is minimal marker migration between day 0 and day 7 CT scans, leading us to perform marker placement and simulation on the same day.^17^ To our knowledge, no patients had meaningful migration of their markers over the course of therapy. If migration did occur, this could have caused inaccurate treatment targeting and potentially resulted in greater shift frequency. Although prior studies have examined treatment time in relation to IFM, we did not control for treatment time in our reporting of the data, which may influence the reporting of our shift frequency. The vast majority of patients, however had either two or three arcs of therapy, so the range of treatment time is relatively narrow. There was a manual aspect to the identification and correction of IFM which may have influenced our findings, in comparison to a fully automated process of IFM correction. Bladder filling was controlled through catheterization and instillation of 120 cc at simulation, and rectal volume was controlled with an enema (for fiducial marker placement) so any patient variance in bladder or rectal volume were not able to be studied as a factor influencing IFM. We did not routinely obtain CBCTs after observing IFM to be able to comment on the potential contribution and consequences of prostate deformation or variation in bladder and rectal filling on dosimetry. Thus, we cannot conclude whether bladder or rectal filling plays the more dominant role in IFM. We also did not require patients to follow strict bladder or rectal filling instructions prior to treatment, even though men did have bladder and rectal volume regulated at simulation. This discrepancy may have potentially contributed towards some variance in interfraction setup, and it is unclear whether it would have also affected intrafraction setup error.

## 5 CONCLUSIONS

IFM occurs in a significant percentage of men undergoing prostate RT with VMAT, and is correctable utilizing a commercially available tracking feature. On MVA, rectal width and volume were associated with a larger SP. Treatment approaches that do not account for IFM should consider including methods of immobilization, improved image guidance, or larger PTV margins in order to avoid marginal miss of the prostate, which can be increasingly consequential with shorter courses of treatment.

## AUTHOR CONTRIBUTIONS

**Lucas M. Serra**: Methodology; formal analysis; data curation; writing—original draft; writing—review & editing. **Tianming Wu**: Methodology; formal analysis; resources; data curation; writing—review & editing. **Mark C. Korpics**: Resources; writing—review & editing. **Kamil Yenice**: Data curation; writing—review & editing. **Stanley L. Liauw**: Conceptualization; methodology; formal analysis; resources; data curation; writing—original draft; writing—review & editing.

## CONFLICT OF INTEREST STATEMENT

The authors declare no conflicts of interest.

## Supporting information



Supporting Information

## References

[1] Shimomura A , Wu T , Rusu I , et al. Monitoring intrafraction motion of the prostate during radiation therapy: suggested practice points from a focused review. Pract Radiat Oncol. 2023:S1879850023002795. 10.1016/j.prro.2023.08.017 37875222

[2] Eastham JA , Auffenberg GB , Barocas DA , et al. Clinically localized prostate cancer: AUA/ASTRO guideline. Part III: principles of radiation and future directions. J Urol. 2022;208(1):26‐33. 35536141 10.1097/JU.0000000000002759

[3] Korpics MC , Rokni M , Degnan M , Aydogan B , Liauw SL , Redler G . Utilizing the TrueBeam Advanced Imaging Package to monitor intrafraction motion with periodic kV imaging and automatic marker detection during VMAT prostate treatments. J Appl Clin Med Phys. 2020;21(3):184‐191. 31981305 10.1002/acm2.12822 PMC7075383

[4] Hsu A , Pawlicki T , Luxton G , Hara W , King CR . A study of image‐guided intensity‐modulated radiotherapy with fiducials for localized prostate cancer including pelvic lymph nodes. Int J Radiat Oncol. 2007;68(3):898‐902. 10.1016/j.ijrobp.2007.02.030 17459610

[5] Gay HA , Barthold HJ , O'Meara E , et al. Pelvic normal tissue contouring guidelines for radiation therapy: a Radiation Therapy Oncology Group consensus panel atlas. Int J Radiat Oncol Biol Phys. 2012;83(3):e353‐e362. 22483697 10.1016/j.ijrobp.2012.01.023 PMC3904368

[6] Aubry JF , Beaulieu L , Girouard LM , et al. Measurements of intrafraction motion and interfraction and intrafraction rotation of prostate by three‐dimensional analysis of daily portal imaging with radiopaque markers. Int J Radiat Oncol. 2004;60(1):30‐39. 10.1016/j.ijrobp.2004.02.045 15337537

[7] Kotte ANTJ , Hofman P , Lagendijk JJW , Van Vulpen M , Van Der Heide UA . Intrafraction motion of the prostate during external‐beam radiation therapy: analysis of 427 patients with implanted fiducial markers. Int J Radiat Oncol. 2007;69(2):419‐425. 10.1016/j.ijrobp.2007.03.029 17513059

[8] Huang E , Dong L , Chandra A , et al. Intrafraction prostate motion during IMRT for prostate cancer. Int J Radiat Oncol. 2002;53(2):261‐268. 10.1016/s0360-3016(02)02738-4 12023128

[9] Wong JR , Grimm L , Uematsu M , et al. Image‐guided radiotherapy for prostate cancer by CT–linear accelerator combination: prostate movements and dosimetric considerations. Int J Radiat Oncol. 2005;61(2):561‐569. 10.1016/j.ijrobp.2004.06.010 15667979

[10] Susil RC , McNutt TR , DeWeese TL , Song D . Effects of prostate‐rectum separation on rectal dose from external beam radiotherapy. Int J Radiat Oncol. 2010;76(4):1251‐1258. 10.1016/j.ijrobp.2009.07.1679 PMC3115781 19939577

[11] Li HS , Chetty IJ , Enke CA , et al. Dosimetric consequences of intrafraction prostate motion. Int J Radiat Oncol. 2008;71(3):801‐812. 10.1016/j.ijrobp.2007.10.049 18234439

[12] Lovelock DM , Messineo AP , Cox BW , Kollmeier MA , Zelefsky MJ . Continuous monitoring and intrafraction target position correction during treatment improves target coverage for patients undergoing SBRT prostate therapy. Int J Radiat Oncol. 2015;91(3):588‐594. 10.1016/j.ijrobp.2014.10.049 25680601

[13] Kisivan K , Antal G , Gulyban A , et al. Triggered imaging with auto beam hold and pre‐/posttreatment CBCT during prostate SABR: analysis of time efficiency, target coverage, and normal volume changes. Pract Radiat Oncol. 2021;11(2):e210‐e218. 32454177 10.1016/j.prro.2020.04.014

[14] Adamson J , Wu Q . Inferences about prostate intrafraction motion from pre‐ and posttreatment volumetric imaging. Int J Radiat Oncol. 2009;75(1):260‐267. 10.1016/j.ijrobp.2009.03.007 PMC2730426 19515507

[15] Roch M , Zapatero A , Castro P , et al. Impact of rectum and bladder anatomy in intrafractional prostate motion during hypofractionated radiation therapy. Clin Transl Oncol. 2019;21(5):607‐614. 30328558 10.1007/s12094-018-1960-y

[16] Butler WM , Merrick GS , Reed JL , Murray BC , Kurko BS . Intrafraction displacement of prone versus supine prostate positioning monitored by real‐time electromagnetic tracking. J Appl Clin Med Phys. 2013;14(2):198‐208. 10.1120/jacmp.v14i2.4141 PMC5714377 23470943

[17] Kumar KA , Wu T , Tonlaar N , Stepaniak C , Yenice KM , Liauw SL . Image‐guided radiation therapy for prostate cancer: a computed tomography–based assessment of fiducial marker migration between placement and 7 days. Pract Radiat Oncol. 2015;5(4):241‐247. 25543198 10.1016/j.prro.2014.11.007

[18] Shelton J , Rossi PJ , Chen H , Liu Y , Master VA , Jani AB . Observations on prostate intrafraction motion and the effect of reduced treatment time using volumetric modulated arc therapy. Pract Radiat Oncol. 2011;1(4):243‐250. 24674002 10.1016/j.prro.2011.02.008

[19] Rose C , Ebert MA , Mukwada G , Skorska M , Gill S . Intrafraction motion during CyberKnife® prostate SBRT: impact of imaging frequency and patient factors. Phys Eng Sci Med. 2023;46(2):669‐685. 36971949 10.1007/s13246-023-01242-7

[20] De Muinck Keizer DM , Pathmanathan AU , Andreychenko A , et al. Fiducial marker based intra‐fraction motion assessment on cine‐MR for MR‐linac treatment of prostate cancer. Phys Med Biol. 2019;64(7):07NT02. 10.1088/1361-6560/ab09a6 30794995

[21] De Muinck Keizer DM , Kerkmeijer LGW , Willigenburg T , et al. Prostate intrafraction motion during the preparation and delivery of MR‐guided radiotherapy sessions on a 1.5T MR‐Linac. Radiother Oncol. 2020;151:88‐94. 32622779 10.1016/j.radonc.2020.06.044

[22] Schröder F , Crawford ED , Axcrona K , Payne H , Keane TE . Androgen deprivation therapy: past, present and future. BJU Int. 2012;109(s6):1‐12. 10.1111/j.1464-410X.2012.11215.x 22672120

[23] Kucway R , Vicini F , Huang R , Stromberg J , Gonzalez J , Martinez A . Prostate volume reduction with androgen deprivation therapy before interstitial brachytherapy. J Urol. 2002;167(6):2443‐2447. 11992054

[24] Langenhuijsen JF , Van Lin EN , Hoffmann AL , et al. Neoadjuvant androgen deprivation for prostate volume reduction: the optimal duration in prostate cancer radiotherapy. Urol Oncol Semin Orig Investig. 2011;29(1):52‐57. 10.1016/j.urolonc.2009.03.024 19523856

[25] Choi H , Chung H , Park JY , Lee JG , Bae JH . The influence of androgen deprivation therapy on prostate size and voiding symptoms in prostate cancer patients in Korea. Int Neurourol J. 2016;20(4):342‐348. 28043112 10.5213/inj.1632628.314 PMC5209578

