# Supplementary material for: Online correction of intrafraction motion during volumetric modulated arc therapy for prostate radiotherapy using fiducial‐based kV imaging: A cohort study quantifying the frequency of shifts and analysis of men at highest risk
Source: J Appl Clin Med Phys. 2025 Jan 17;26(4):e14603. doi: 10.1002/acm2.14603 (PMC11969104; doi:10.1002/acm2.14603)

**Supplemental figures**

Supplemental Figure 1A, 1B, and 1C: Histograms of Left/Right, Anterior/Posterior and Superior/Inferior

Supplemental Figure 2. Linear fit of rectal width and shift percent, according to use of androgen deprivation therapy (ADT).


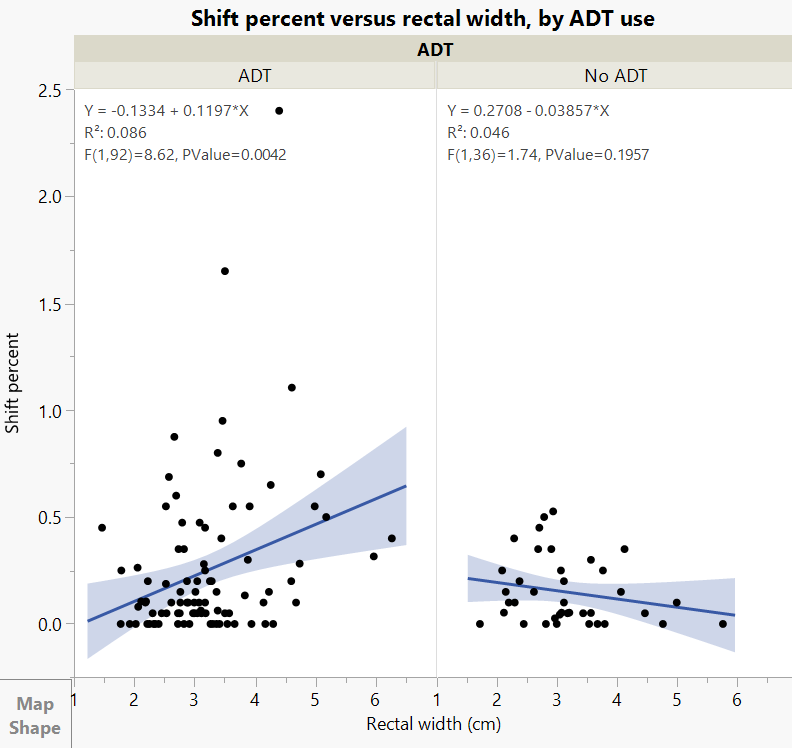

Supplement: Supplementary file 1 — Supporting Information [file ACM2-26-e14603-s001.docx]
